# Formation and Physico-Chemical Evaluation of Nifedipine-hydroxypropyl-β-cyclodextrin and Nifedipine-methyl-β-cyclodextrin: The Development of Orodispersible Tablets

**DOI:** 10.3390/ph15080993

**Published:** 2022-08-12

**Authors:** Emma Adriana Ozon, Marian Novac, Daniela Gheorghe, Adina Magdalena Musuc, Mirela Adriana Mitu, Iulian Sarbu, Valentina Anuta, Adriana Rusu, Simona Petrescu, Irina Atkinson, Dumitru Lupuliasa

**Affiliations:** 1 Department of Pharmaceutical Technology and Biopharmacy, Faculty of Pharmacy, “Carol Davila” University of Medicine and Pharmacy, 6 Traian Vuia Street, 020945 Bucharest, Romania; 2 “Ilie Murgulescu” Institute of Physical Chemistry, 202 Spl. Independentei, 060021 Bucharest, Romania; 3 Department of Pharmaceutical Physics and Biophysics, Drug Industry and Pharmaceutical Biotechnologies, Faculty of Pharmacy, “Titu Maiorescu” University, 004051 Bucharest, Romania; 4 Department of Physical and Colloidal Chemistry, Faculty of Pharmacy, “Carol Davila” University of Medicine and Pharmacy, 020956 Bucharest, Romania

**Keywords:** nifedipine, inclusion complexes, hydroxypropyl-β-cyclodextrin, methyl-β-cyclodextrin orodispersible tablets

## Abstract

The novelty in this study is the development of new orodispersible tablets containing nifedipine (NIF) as the active ingredient. Initially, the formation of inclusion complexes between nifedipine and two derivatives of beta-cyclodextrin, namely, hydroxypropyl-β-cyclodextrin (HP-β-CD) and methyl-β-cyclodextrin (Me-β-CD), was established. Inclusion complexes of nifedipine were prepared by different procedures: kneading, coprecipitation and lyophilization methods, using a 1:1 molar ratio among the drug and cyclodextrin compounds. A physical mixture was also developed for comparison, with the same molar ratio. The physicochemical and structural properties of these obtained complexes were subsequently analysed using Fourier-transform infrared spectroscopy, scanning electron microscopy, differential scanning calorimetry and X-ray diffraction techniques. The lyophilization method of preparation leads to obtaining the complete inclusion of nifedipine in the used cyclodextrin cavity, for both the derivative cyclodextrins. After that, preformulation studies and manufacturing of orodispersible tablets containing NIF-HP-β-CD and NIF-Me-β-CD, respectively, inclusion complexes were advanced. The obtained findings show that only F3 (which contains NIF-HP-β-CD) and F6 (which contains NIF-Me-β-CD) have a suitable flowability for the direct compression materials.

## 1. Introduction

**Figure 1:**
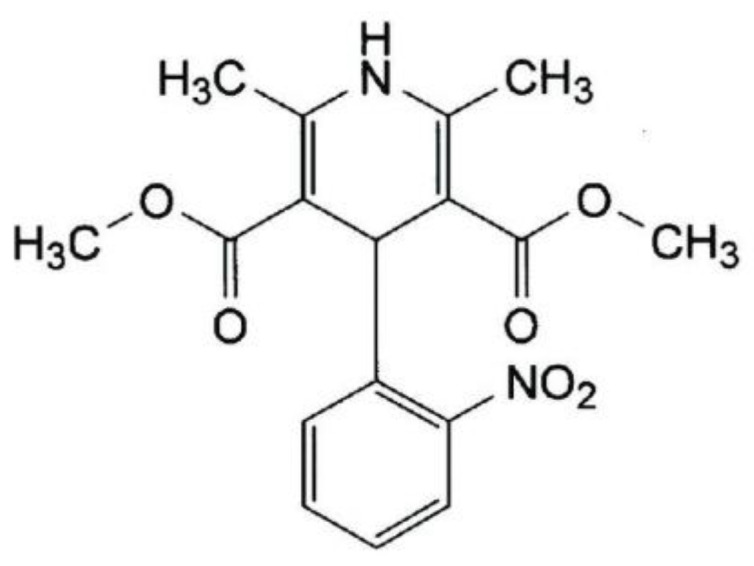
Chemical structure of nifedipine.

Nowadays, one of the most crucial goals of pharmaceutical research is to enhance the solubility of poorly water-soluble drugs [1,2]. The development of partial or full inclusion complexes with cyclodextrins (cyclic oligosaccharides produced by the enzymatic degradation of starch) has been related to improving the dissolution rate, the solubility and, therefore, the bioavailability in poorly water-soluble drugs [3,4,5,6]. The most valuable characteristic of cyclodextrin compounds is that they can encapsulate the drug in their hydrophobic internal cavity [7], for improving their physicochemical properties. Nifedipine (dimethyl 2,6–dimethyl–4–(2–nitrophenyl)–1,4–dihydropyridine–3,5–dicarboxylate, Figure 1) is a dihydropyridine calcium-channel-blocking agent that is classified as a poorly water-soluble drug. This is associated with poor dissolution characteristics and, therefore, with poor oral bioavailability and it possesses high permeability, nifedipine belonging to the biopharmaceutical classification system (BCS) as a class II substance. Nifedipine has the most important characteristic to inhibit the transmembrane influx of extracellular calcium ions into myocardial and vascular smooth muscle cells and, by these, to cause a prolonged vasodilatation of the coronary and systemic arteries and to decrease the myocardial contractility [8].

Until now, various approaches to enhance the dissolution rate of nifedipine have been reported: the compaction process using hydroxypropylmethylcellulose [9], co-grinding methods using HPMC, a water-soluble polymer [10] or sodium deoxycholate [11], the formation of solid dispersions prepared by hot-melt methods, such as co-precipitates or co-evaporates with mannitol [12], with phosphatidyl choline esters [13], with HPMC [14], with chitosan and chitosan glutamate [15], with polyethylene glycol 6000 [16] and with polyoxyethylene–polyoxypropylene copolymers [17], as well as the formation of inclusion complexes with various cyclodextrins [18,19,20,21]. Another study showed an enhancement in the solubility and dissolution rate of the physical mixtures of nifedipine with SEPITRAP 80 (263% increase of the solubility) and with SEPITRAP 4000 (368% increase of the solubility), respectively [22].

In this work, hydroxypropyl-β-cyclodextrin and methyl-β-cyclodextrin were individually used as the host molecules and nifedipine as the guest molecule. The inclusion complexes were prepared by kneading, coprecipitation and lyophilization methods in a 1:1 molar ratio. The structures, morphologies and chemical properties of the inclusion complexes obtained using different techniques, as well as the simple physical mixture in the same molar ratio, were discussed. The novelty in this study consists of the preformulating and manufacturing processes of the nifedipine inclusion complexes obtained using the lyophilization method for both used cyclodextrin in new oral dispersible tablets.

## 2. Results

### 2.1. Binary System Characterization

All binary systems obtained were off-white, uniform and fine powders, with different appearances based on the obtaining process.

### 2.2. Structural, Morphological and Chemical Characterization

**Figure 2:**
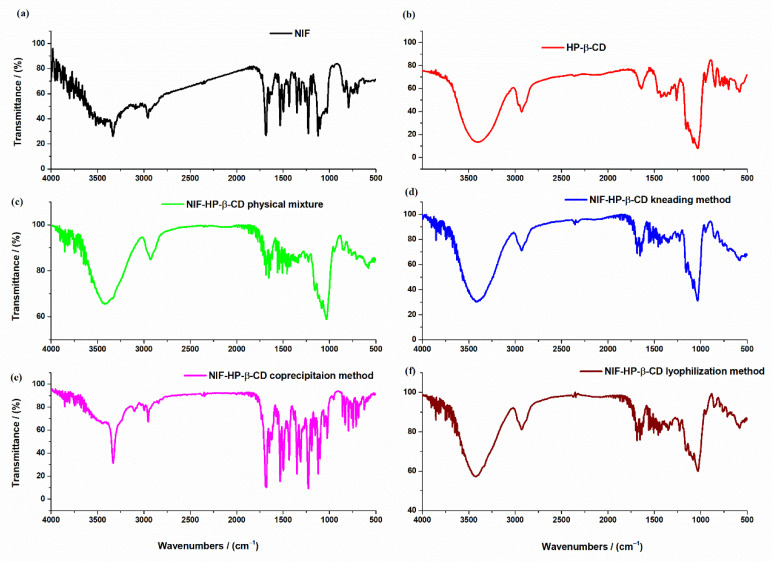
FTIR spectra of (**a**) NIF; (**b**) HP-β-CD; (**c**) physical mixture between NIF and HP-β-CD; and inclusion complexes (**d**) NIF-HP-β-CD kneading technique; (**e**) NIF-HP-β-CD coprecipitation technique and (**f**) NIF-HP-β-CD lyophilization technique.

FTIR spectra of NIF, HP-β-CD, NIF-HP-β-CD physical mixture and NIF-HP-β-CD inclusion complexes obtained using the kneading, coprecipitation and lyophilization methods are represented in Figure 2. The FTIR technique was employed to evaluate the possible interaction of nifedipine with the two used β-cyclodextrin derivatives: HP-β-CD and Me-β-CD, respectively, in their physical mixture and inclusion complexes obtained using kneading, coprecipitation and lyophilization methods.

The main characteristic peaks from the FTIR spectra related to functional groups are shown in Table 1.

The main FTIR absorption peaks of pure nifedipine (Figure 2a) are at 3334 cm^−1^ due to NH stretching, at 1689 cm^−1^ assigned to C=O ester bond, at 1624 cm^−1^ characteristic to C=C aromatic vibration, at 1529 cm^−1^ due to NO_2_ and at 1122 cm^−1^ due to -C-O ester [23,24]. The FTIR spectrum of HP-β-CD (Figure 2b) showed the main characteristics of IR absorption peaks belonging to saccharides: the wide and strong absorption bond at 3409 cm^−1^ due to O-H stretching vibration caused by the intramolecular hydrogen bond, at 2925 cm^−1^ due the anti-symmetric vibration of methyl groups (C-H stretching vibration), at 1640 cm^−1^ due to O-H bending vibration and 1157 cm^−1^ due to C-O vibration [25,26]. The characteristic absorption peak of α-type glycosidic bond found at 846 cm^−1^ indicates the formation of HP-β-CD by glucopyranose units through α-1,4-glycosidic bond [27]. In the FTIR spectra of NIF-HP-β-CD physical mixture (Figure 2c) and NIF-HP-β-CD obtained using coprecipitation methods (Figure 2e), no new peaks were observed. The main peaks correlated to the functional groups of the two compounds were kept. In the case of NIF-HP-β-CD inclusion compounds prepared by the kneading (Figure 2d) and lyophilization (Figure 2f) methods, a shift in main absorption bands of NIF and HP-β-CD was observed. These findings indicate the involvement of these functional groups in some interactions related to the formation of the inclusion complexes.

**Figure 3:**
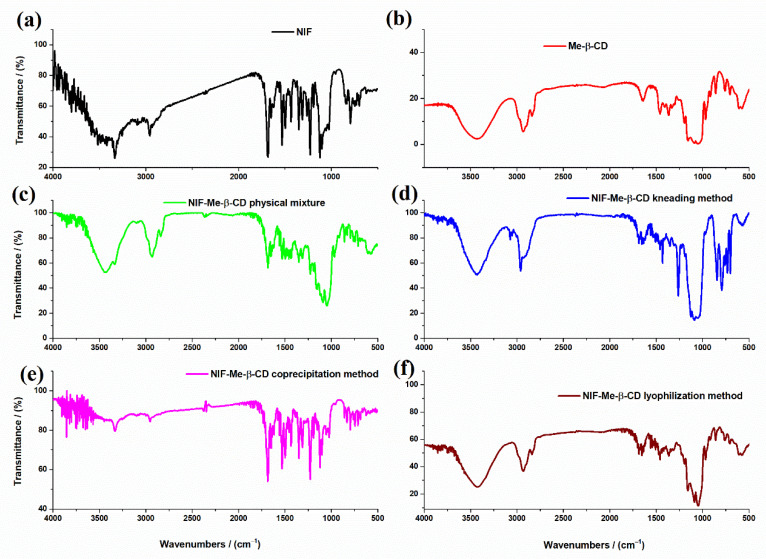
FTIR spectra of (**a**) NIF; (**b**) Me-β-CD; (**c**) physical mixture of NIF and Me-β-CD; and inclusion complexes (**d**) NIF-Me-β-CD kneading technique; (**e**) NIF-Me-β-CD coprecipitation technique and (**f**) NIF-Me-β-CD lyophilization technique.

FTIR spectra of NIF, Me-β-CD, their physical mixture and NIF-Me-β-CD inclusion complexes obtained using the kneading, coprecipitation and lyophilization methods are represented in Figure 3.

The main characteristic peaks from the FTIR spectra related to functional groups are shown in Table 2.

The FTIR spectrum of Me-β-CD (Figure 3b) shows the main characteristic bands belonging to saccharides. The FTIR peaks at 2933 cm^−1^ and 2840 cm^−1^ are due to the aliphatic C-H region of Me-β-CD. The stretching frequency of the C-OH primary and secondary groups from Me-β-CD molecule appears at approximately 1029 cm^−1^ [28,29,30]. There are no new peaks found in the spectra of NIF-Me-β-CD physical mixture (Figure 3c) and the inclusion complexes obtained using different methods of complexation: kneading (Figure 3d), coprecipitation (Figure 3e) and lyophilization (Figure 3f). Some band characteristics of NIF do not appear in any inclusion complexes, even in the physical mixture of NIF-Me-β-CD (Figure 3c) where no complexation is expected. It is possible that those bands are masked by the very intense and broad bands of the Me-β-CD within the same wavelength region (2960 cm^−1^, 1682 cm^−1^ and 1122 cm^−1^). These bands are visible only in the FTIR spectrum of the compound obtained using the coprecipitation method (Figure 3e). The disappearance or the strong reduction in the characteristic bands of NIF from the FTIR spectra of compounds NIF-Me-β-CD obtained by kneading (Figure 3d) and lyophilization (Figure 3f) methods is an indication of strong interactions between NIF and cyclodextrin compounds and the possible complexation by the inclusion of the drug into the Me-β-CD cavity. All these changes observed in the FTIR spectra of NIF-HP-β-CD and NIF-Me-β-CD compounds obtained using physical and chemical methods of complexation, such as the shift in the absorption peaks or their decrease in intensity, even the whole disappearance is evidence of the various degrees of interaction or amorphization in different products [28].

SEM analysis of the pure and inclusion complexes is usually used to characterize the structural aspects of the raw compounds and the obtained products but is an inadequate technique for confirming the complexation process. SEM analysis will help us to prove the existence of a single component in the final obtained product [31].

**Figure 4:**
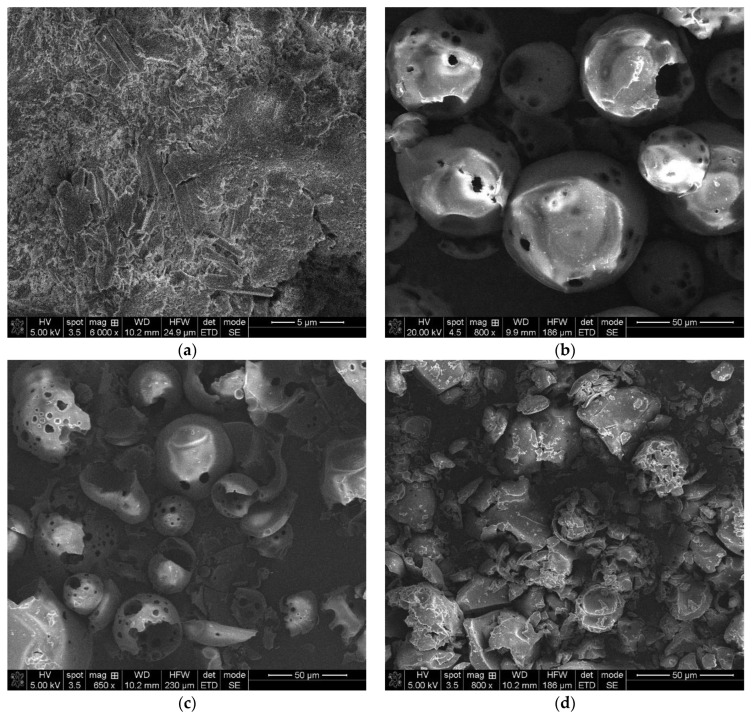

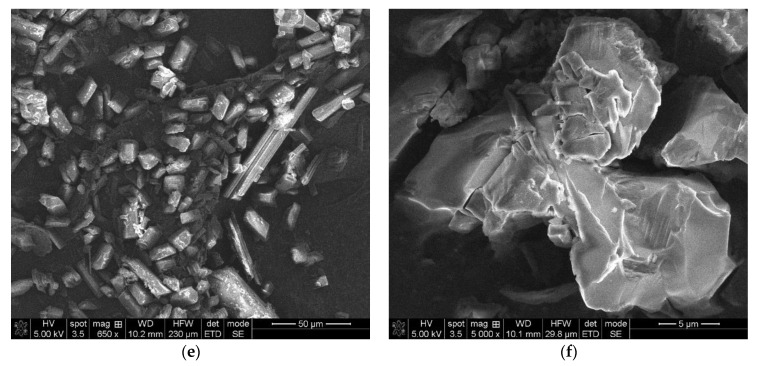
SEM images of (**a**) NIF; (**b**) HP-β-CD; (**c**) the physical mixture between NIF and HP-β-CD; and inclusion complexes (**d**) NIF-HP-β-CD kneading technique; (**e**) NIF-HP-β-CD coprecipitation technique and (**f**) NIF-HP-β-CD lyophilization technique.

The morphologies of NIF, HP-β-CD, the physical mixture of the two compounds and the NIF-HP-β-CD inclusion complexes obtained using kneading, coprecipitation and lyophilization methods are represented in Figure 4. The SEM images of the compounds under study were obtained by scanning the entire surface of the studied samples and magnification factors of 500 until ×3500. The most representative SEM images, which provide significant information regarding the morphologies of the studied samples, are considered. NIF particles (Figure 4a) show variable shape with a rough surface and showing loose aggregates of irregular shape [12]. SEM images of HP-β-Cd show spherical forms of particles (Figure 4b). The SEM image of NIF-HP-β-Cd physical mixture (Figure 4c) obviously shows the NIF characteristics mixed with HP-β-Cd particles, which clearly displays the presence of the drug in the physical mixture. A drastic modification in the morphologies was observed in the products obtained using kneading, coprecipitation and lyophilization methods. In these compounds, it is not possible to distinguish between the two compounds, revealing a possible interaction between the NIF and HP-β-CD in those systems. In the lyophilization process (Figure 4f), the formation of a new solid phase with an evident loss of crystallinity can be detected, with respect to the initial compounds.

**Figure 5:**
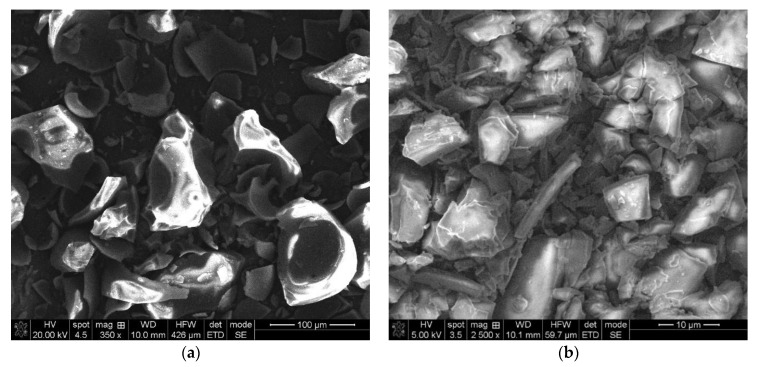

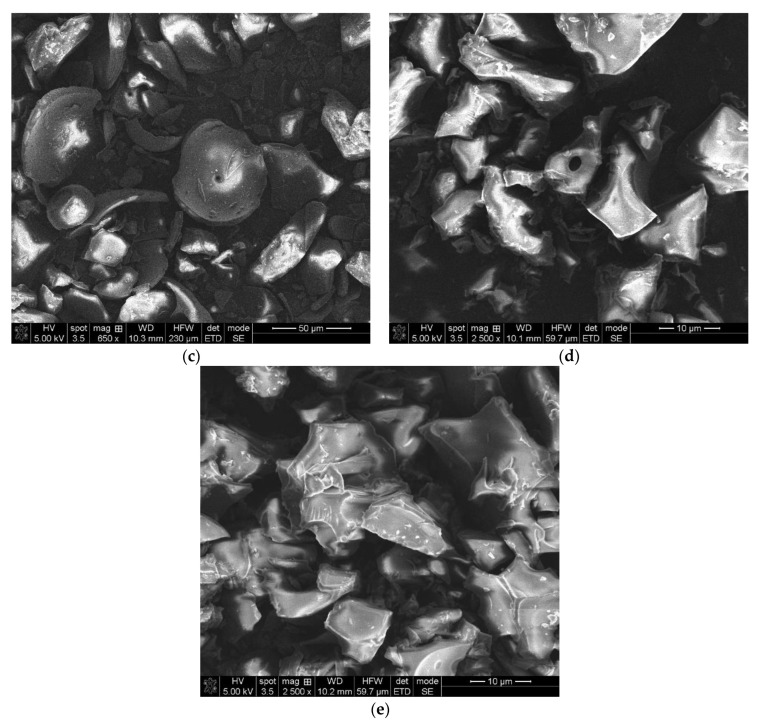
SEM images of (**a**) Me-β-CD; (**b**) physical mixture between NIF and Me-β-CD; and inclusion complexes (**c**) NIF-Me-β-CD kneading technique; (**d**) NIF-Me-β-CD coprecipitation technique and (**e**) NIF-Me-β-CD lyophilization technique.

The morphologies of Me-β-CD, the physical mixture and the NIF-Me-β-CD inclusion complexes obtained using kneading, coprecipitation and lyophilization methods are represented in Figure 5. Me-β-CD (Figure 5a) shows an amorphous morphology, composed of spherical particles, some of them being fragments of spherical shells [32,33].

The NIF-Me-β-CD physical mixture (Figure 5a) shows the presence of characteristic NIF crystals in a mixture with Me-β-CD particles or some of them adhered to the Me-β-CD particle surface. In the NIF-Me-β-CD kneading product (Figure 5c), even if one can detect the development of a new phase, it is still possible to detect some isolated methyl-β-cyclodextrin particles. The coprecipitation technique (Figure 5d) produced some amorphous aggregates. The SEM image of the NIF-Me-β-CD inclusion product obtained using the lyophilization method shows an amorphous aspect of a new solid phase, demonstrating the possible interaction between the NIF and Me-β-CD by the inclusion of the drug in the cyclodextrin cavity.

**Figure 6:**
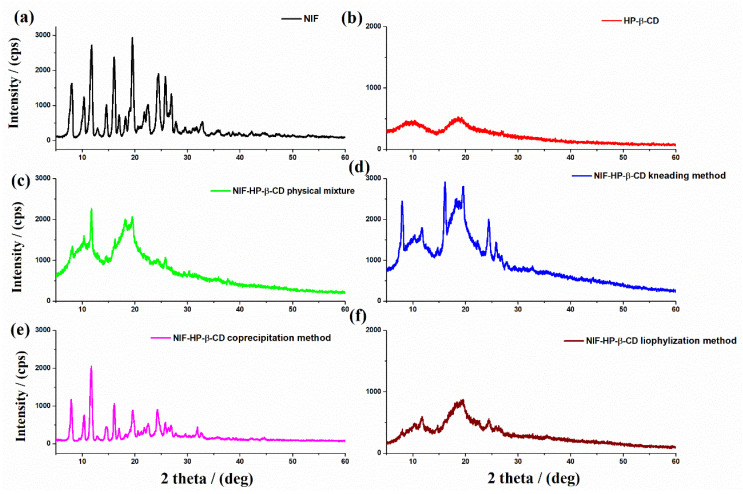
X-ray diffraction patterns of (**a**) NIF; (**b**) HP-β-CD; (**c**) physical mixture between NIF and HP-β-CD; and inclusion complexes (**d**) NIF-HP-β-CD kneading technique; (**e**) NIF-HP-β-CD coprecipitation technique and (**f**) NIF-HP-β-CD lyophilization technique.

X-ray powder diffraction is a suitable technique that clearly shows the cyclodextrin complexation. A true inclusion complex is formed when a new XRD pattern is obtained and is different from that of the superimposition of each pure component [34]. The X-ray diffraction patterns of NIF, HP-β-CD, their physical mixture and NIF-HP-β-CD obtained using kneading, coprecipitation and lyophilization methods are shown in Figure 6. The X-ray diffraction pattern of pure nifedipine (Figure 6a) exhibited characteristic diffraction peaks at 2θ = 8.1, 11.8, 16.6, 19.5 and 24.5°, which are attributed to the crystal planes of Miller indices of (100), (002), (200), (211) and (300) [35]. This nifedipine diffractogram corresponds to crystalline polymorph A [18]. The XRD pattern of HP-β-CD (Figure 6b) showed its amorphous structure with broad peaks around 2θ = 9.76° and 18.7°. In the binary system, prepared by a simple physical mixture (Figure 6c) and also by the coprecipitation method (Figure 6e), the presence of the characteristic peaks of the pure NIF and HP-β-CD compounds are observed. This is a confirmation that the complexation between the two compounds did not occur. A possible interaction between them is still evidenced due to the reduction in the intensities of the diffraction peaks. In contrast, in the NIF-HP-β-CD systems obtained using kneading (Figure 6d) and lyophilization (Figure 6f) methods, a reduction in the crystallinity degree of the obtained products was noted, even though some specific XRD peaks corresponding to the NIF compound are still noticeable. The additional sharp peaks of NIF indicate that the inclusion is only partial due to the presence of a non-included drug molecule.

**Figure 7:**
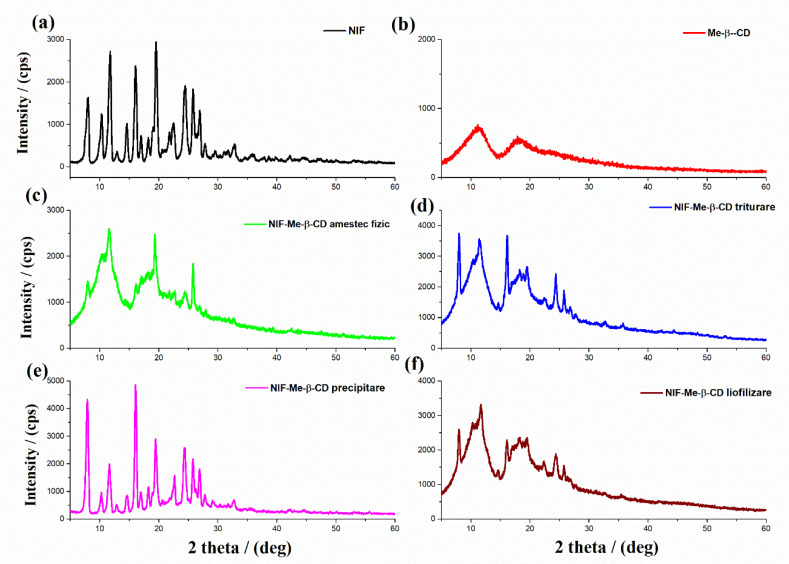
X-ray diffraction patterns of (**a**) NIF; (**b**) Me-β-CD; (**c**) NIF-Me-β-CD physical mixture; and inclusion complexes (**d**) NIF-Me-β-CD kneading technique; (**e**) NIF-Me-β-CD coprecipitation technique and (**f**) NIF-Me-β-CD lyophilization technique.

The X-ray diffraction patterns of NIF, Me-β-CD, NIF-Me-β-CD physical mixture and NIF-Me-β-CD obtained using kneading, coprecipitation and lyophilization methods are shown in Figure 7. The XRD diffractogram of Me-β-CD (Figure 7b) revealed a hollow pattern, which is an indication of its amorphous character. Comparing the XRD diffraction patterns of pure compounds and their NIF-Me-β-CD physical mixture (Figure 7c), it is observed that the X-ray diffractogram of the physical mixture is a combination of the two initial components, with a slight decrease in the intensity of the diffraction peaks. The coprecipitation system (Figure 7e) showed an X-ray diffraction pattern relatively comparable to that achieved by combining the diffraction peaks of pure compounds. However, the observed lower intensity of its diffraction peaks can be described by the reciprocal interactions in the liquid state. The kneading (Figure 7d) and lyophilization (Figure 7f) products displayed a complete amorphous character, which confirms the strong ability of the amorphous Me-β-CD compound to induce the NIF amorphization [36]. Further, the lyophilization process can induce a loss of the crystallinity state and the existence of a new solid phase proved the formation of the inclusion complex in the 1:1 molar ratio between NIF and Me-β-CD [37]. The XRD pattern of the complex obtained using the lyophilization procedure confirmed the inclusion of the NIF compound into the amorphous lattice of the “host” cyclodextrin, since it retained the characteristic XRD pattern of the “host” molecule.

**Figure 8:**
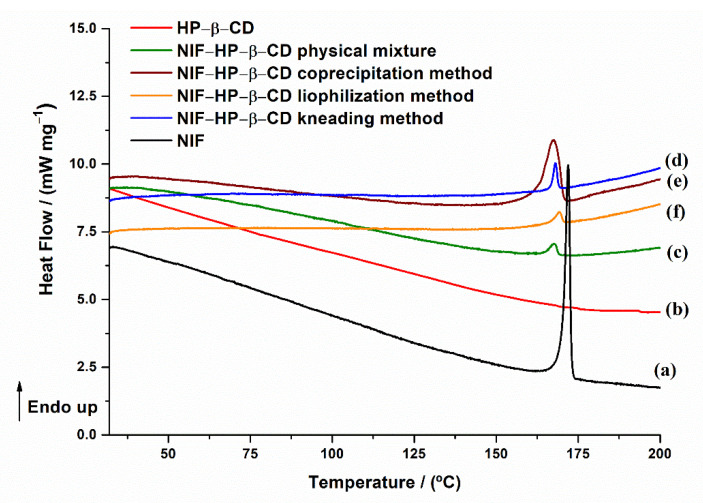
DSC curves of (**a**) NIF; (**b**) HP-β-CD; (**c**) NIF-HP-β-CD physical mixture; and inclusion complexes (**d**) NIF-HP-β-CD kneading technique; (**e**) NIF-HP-β-CD coprecipitation technique and (**f**) NIF-HP-β-CD lyophilization technique.

The thermal properties of the pure compounds and their physical mixtures and the products obtained using kneading, coprecipitation and lyophilization methods of preparation were evaluated using differential scanning calorimetry. The DSC thermal curves of NIF, HP-β-CD, their physical mixture and the inclusion complexes are depicted in Figure 8.

The DSC curve of pure NIF showed a sharp endothermic peak at *T*_min_~172 ± 0.7 °C, ascribed to the melting process of the pure compound, with a melting enthalpy of Δ*H* = 99.07 ± 0.32 Jg^−1^. The DSC curve of HP-β-CD showed a slight decrease in the slope of the curve from room temperature to 130 °C, which could be related to the slow release from the cyclodextrin cavity of the crystallized water molecules [38,39]. The DSC curve of the NIF-HP-β-CD physical mixture (Figure 8c) shows a slight decrease in the peak temperature at *T*_min_ = 168 ± 0.9 °C (Δ*H* = 7.87 ± 0.6 Jg^−1^). These results indicate that the two compounds were physically mixed with some interactions. The products obtained using the kneading, coprecipitation and lyophilization methods exhibited a higher decrease in the peak melting intensity of NIF and a shift of peak temperatures at lower values, even its disappearance in the case of the lyophilization technique (Figure 8f), which indicates an interaction between the two components. The DSC results indicated that NIF was successfully incorporated into the HP-β-CD cavity in the case of the lyophilization product. Generally, when guest molecules are partially or totally included in the cyclodextrin cavity, their sublimation, boiling or melting temperatures are shifted to different temperatures or disappear [40,41,42].

**Figure 9:**
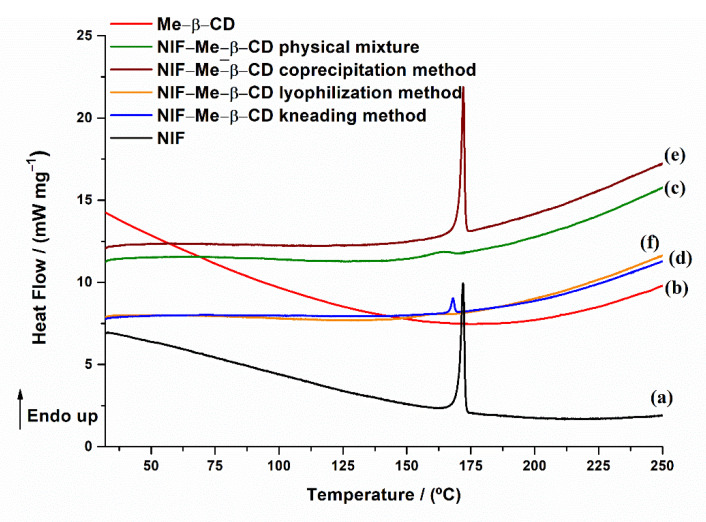
DSC curves of (**a**) NIF; (**b**) Me-β-CD; (**c**) NIF-Me-β-CD physical mixture; and inclusion complexes (**d**) NIF-Me-β-CD kneading technique; (**e**) NIF-Me-β-CD coprecipitation technique and (**f**) NIF-Me-β-CD lyophilization technique.

The DSC curves of NIF, Me-β-CD, the physical mixture between NIF and Me-β-CD and the inclusion complexes are illustrated in Figure 9.

The DSC curve of Me-β-CD (Figure 9b) shows no thermal effects until 250 °C, when the sample is heated using a linear heating rate of 10 °C min^−1^. In the coprecipitation system (Figure 9e), the melting peak characteristic of the NIF is maintained, indicating that no complexation occurs between NIF and Me-β-CD in the 1:1 molar ratio in this system. Regarding the kneading (Figure 9d) and lyophilization (Figure 9f) systems, a considerable peak intensity reduction and a shift to a lower temperature of the melting peak temperature of NIF (*T*_min_ = 168 °C), even disappearance in the case of lyophilization, were observed. Compared with the physical mixture, it can be pointed out that some NIF-Me-β-CD interactions exist. The absence of the NIF melting peak in the lyophilization product implies the formation of the inclusion complex because of the encapsulation of the drug into the cyclodextrin cavity [28].

### 2.3. Preformulation Studies for the Orodispersible Tablets, Which Contain NIF-HP-β-CD and NIF-Me-β-CD, Respectively, Inclusion Complexes

When considering the direct compression technology, the particle size of the blend is an important parameter for the powder flowability, die filling and tablets’ uniformity and integrity. It is essential to achieve an optimal particle size distribution, to obtain tablets with satisfactory pharmacotechnical and biopharmaceutical properties [43,44]. The width of the particle size distribution must be established in the preformulation studies.

**Figure 10:**
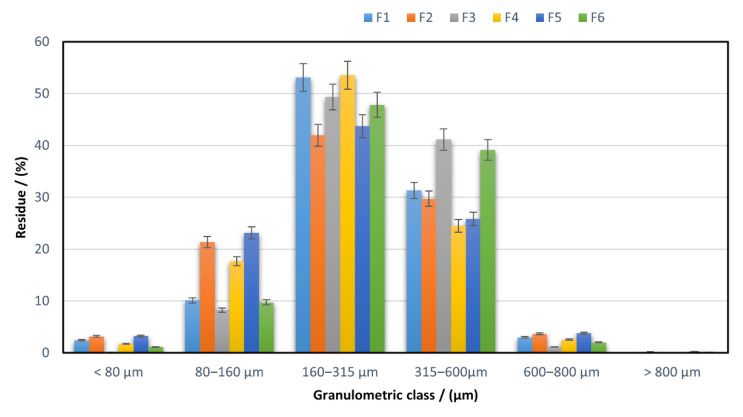
Granulometric study of the materials used for direct compression.

Figure 10 shows a registered histogram by plotting the particle size distribution on granulometric classes in the case of the studied powders.

A narrow particle size distribution is preferable for ensuring good flowability and compressibility characteristics in the blend [45]. The studied formulations display different granulometric properties, with similar behaviour between F3 and F6 on one side and the other four (F1, F2, F4 and F5) on the other side. The materials based on the silicified microcrystalline cellulose and Disintequik™ ODT mixture have a considerable proportion of the particles with sizes between 160 and 600 µm, above 90% for F3 and almost 87% for F6, showing a narrower range for all proposed formulations. Meanwhile, F2 and F5, which contain F-Melt^®^ and Disintequik™ ODT excipients, possess the highest amount of particles in a 80–160 µm range of all formulations. The results prove that the type of cyclodextrin used in the complex formation is not influencing the particle size distribution, but the greatest influence is given by the direct compressible excipient types and amounts.

The pharmacotechnical properties in the direct compression powders are shown in Table 3.

The moisture content is a very important powder property that highly influences both the blend behaviour during the compression process and its consistency and also the quality of the final tablets [46]. Considering that water was used for the preparation of the active ingredients by the lyophilization method, we expected to find a certain amount of moisture in the final blends. The results show there is no significant difference between the batches containing NIF-HP-β-CD and the ones based on NIF-Me-β-CD, proving that the type of cyclodextrin is not influencing the moisture retention in the material. On the other hand, obviously, the direct compression excipient types and amounts induce notable variations between formulations in terms of humidity content. The lowest amounts of moisture were detected in F3 (2.45%) and F6 (2.7%), the samples containing silicified microcrystalline cellulose and Disintequik™ ODT mixture. Meanwhile, the other four formulations enclose double amounts of water, 4.69% in F1, 5.13% in F2, 4.85% in F4 and the highest content of 5.76% is included in F5.

The flowability considerably varies between the formulations, regardless of the type of cyclodextrin used. F3 presents the highest flow rate of 6.185 g/mL, followed by F6 with a 5.825 g/mL flow rate. F1, F2, F4 and F5 registered a longer flow time, almost double compared to F3 and F6. According to the European Pharmacopoeia specifications on the angle of repose values, F3 and F6 are classified as having an excellent free flow, while F2 and F5 present a good flow and F1 and F4 have just a fair flow. Considering that the analysis could not be performed on lower-diameter nozzles as the samples did not flow and the results were recorded for the 15 mm nozzle, it can be admitted that only F3 and F6 possess a suitable flowability for the direct compression materials [47].

The volumetric characteristics proved, more precisely, the differences between the batches and the influence of the cyclodextrin type on the flowability and compressibility parameters. A clear difference between the formulations containing NIF-HP-β-CD complex (F1–F3) and that with NIF-Me-β-CD (F4–F6) was registered. F4–F6 series show higher values for CI and HR than F1–F3 batches that include the same excipients, the cyclodextrin being the only variable. This demonstrates that Me-β-CD decreased the compressibility and flowability properties in the blends. Still, F6 presents a 9.3 CI and 1.10 HR, values which, in accordance with European Pharmacopoeia [48], mean excellent compressibility and flow character. This is due to the chosen excipients and their weight ratio (silicified microcrystalline cellulose and Disintequik™ ODT mixture). F3 recorded the best values for CI (8.6) and HR (1.09) of all formulations, as a result of using the same excipients in the same proportions as F6, plus enclosing the NIF-HP-β-CD complex, which proved to possess better flowing and compressibility properties. In contrast, F2 and F5, the formulations based on F-MELT^®^ and Disintequik™ ODT, exhibited low mechanical attributes, with high values for CI (16.3-F2 and 18.3–F5) and also for HR (1.19–F2 and 1.22–F5). However, the highest values for CI (19.3) and HR (1.24) were revealed for F4, which contains silicified microcrystalline cellulose mixed with F-MELT^®^. Even so, in agreement with the European Pharmacopoeia [48], F1, F2, F4 and F5 have fair flowability and compressibility.

### 2.4. Quality Control of the Orodispersible Tablets

**Figure 11:**
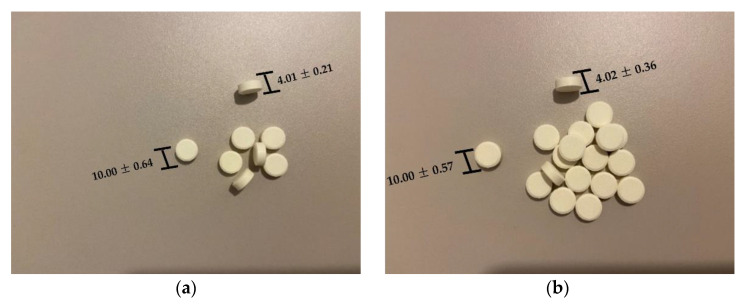
The appearance of the tablets used for oral dispersion ((**a**)-F3 (NIF-HP-β-CD) and (**b**)-F6 (NIF-Me-β-CD)).

The manufactured tablets are round, white yellowish, with homogeneous appearances and smooth surfaces (Figure 11).

The pharmacotechnical characteristics of the obtained tablets are shown in Table 4.

The uniformity of tablet dimensions (thickness and diameter) and weight proves that the dies were homogeneously filled due to the great flowability in the materials, and the compression process was successfully realized based on a good adjustment of all parameters and on the suitable compressibility in the blend [49]. The registered intra- and inter-batch variabilities are low, demonstrating that the type of cyclodextrin is not affecting the tablet dimensions or mass. The thickness of the tablets is around 4 mm, the diameter is 10 mm and the weight is around 300 mg for both formulations. The values met the European Pharmacopoeia requirements [48], revealing the appropriate selection of the excipients and compression conditions.

The mechanical resistance of the tablets is satisfactory, both in terms of hardness and friability, but a clear difference between the formulations is observed, indicating that the type of cyclodextrin is responsible for the changes in tablets’ compactness. Me-β-CD induces a higher hardness and friability (67 N and 0.11%) compared to HP-β-CD (55 N and 0.05%). However, the excipients proved to exhibit suitable plasticity and elasticity properties in the materials, leading to tablets with appropriate mechanical strength for both formulations.

In accordance with the hardness results, the disintegration time in both media for the two formulations proved to be faster for the NIF-HP-β-CD tablets compared to NIF-Me-β-CD tablets. Further, for both batches, the disintegration time in the water (13 s for F3 and 21 s for F6) was much lower than in the simulated saliva medium (26 s for F3 and 34 s for F6). The disintegration behaviour displayed by both formulations meets the specified requirements of the European Pharmacopoeia [48], but the influence of the cyclodextrin type is evidenced. The HP-β-CD inclusion complex led to tablets with lower hardness and faster disintegration performance. All tablets show an excellent disintegration ability due to the superdisintegrant properties of the excipients, characteristic of silicified microcrystalline cellulose, Disintequik™ ODT and, especially, sodium starch glycolate.

**Figure 12:**
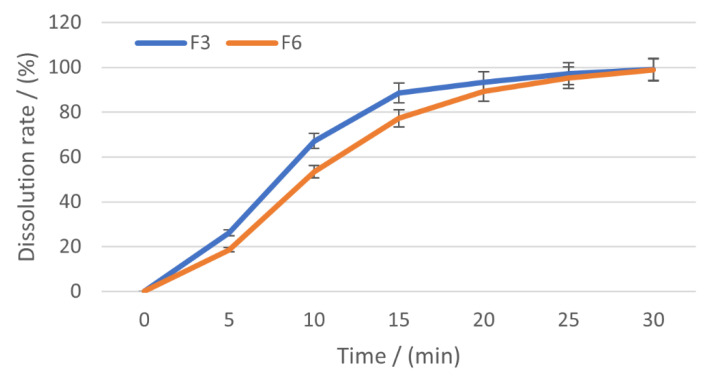
The dissolution behaviour for the orally dispersible tablets.

The dissolution rates registered by the tablets during 30 min on simulated saliva dissolution medium are shown in Table 5 and Figure 12.

After 30 min, the tablets released the entire quantity of nifedipine contained (99.11% for F3 and 98.79% for F6), proving that both formulations lead to fast-dissolving tablets with excellent dissolution rate. Still, the differences between the formulations’ performances are obvious. Even after the first 5 min, it is observed that F3 has a higher dissolution rate of 26.15%, compared to 18.57% for F6, and this behaviour is maintained during the first 20 min, then F6 reaches similar release degrees.

## 3. Discussion

Most of the particles have sizes in the 160–600 µm range, but the variability between the batches marks the differences in the flowing performance, especially for the formulations that contain a higher proportion of particles lower than 160 µm. Excessive fineness is not desirable in the compression process as it can lead to tablet deficiencies, such as capping or lamination, or low-hardness tablets. Still, some authors [50] consider that some fines must fill the interstitial spaces between the larger particles. Kudo Y. et al. [51] stated that diminishing the amount of small particles conduces to improved compressibility. Jonat S. et al. [52] proved that the apparent constant distance is increased by the presence of fine particles on the surfaces and the adhesion forces will decrease. Considering these aspects, the flowability and compressibility behaviour of the formulations can be explained based on the particle size distribution.

Sandler et al. [53] studied the influence of moisture content on the flowability of the powders and demonstrated that in low amounts, it acts as a lubricant, but in high amounts, it increased the cohesion between the particles and considerably reduced the flowing behaviour. The results of this study are in agreement with the related data and it was also noticed that the increased moisture content led to a diminished free flow in the F1, F2, F4 and F5 batches.

The flowing ability of the material is crucial during the compression process and, to ensure tablet uniformity, the powder must flow easily and the dies must be homogenously filled [54]. The major forces between powder particles are friction and cohesion. In order to improve friction, the contact surface must be increased since the particle size influences the links between particles. Simultaneously, the moisture content reduces the friction, but increases the cohesiveness by creating liquid bridges between particles [55]. All these explain the flowing performances of the studied formulations and it seems that only F3 and F6 display desirable flowabilities for the direct compression process.

The parameters that proved the influence of the cyclodextrin types are the volumetric characteristics of the powders expressed as CI and HR. In addition to the cyclodextrin type, the flowability and compressibility in the materials were highly influenced by the selected mixture of direct compression excipients, the moisture content and the particle size distribution. It was proven that the use of HP-β-CD and silicified microcrystalline cellulose and Disintequik™ ODT mixture leads to lower moisture content and narrower particle size distribution, inducing greater flowability and compressibility performances in the materials.

Usuda S. et al. [56] revealed the crucial influence of the compression conditions, including the die-hole position, on tablet weight variation. The recorded results for the tablets sizes and weight indicate that the direct compression process was well conducted in the case of the studied formulations. According to Hashmat D. et al. [57], the variation in weight uniformity of the tablets depends on the compression pressure, the die filling fluctuation and blend characteristics. It is noticed that all these factors were properly established and tablets with adequate and within the limit characteristics were achieved.

Even though the type of cyclodextrin clearly affected the mechanical resistance of the tablets, with higher values for the Nif-Me-β-CD complex, the selection of the excipients and compression force led to optimal-strength tablets. As Olsson H. et al. [58] stated, the mechanical behaviour of the tablets is closely related to their in vivo performance and, based on the obtained results, it may be considered that F3 will display a better dissolution profile and a faster release of the active ingredient after administration. Cabiscol R. et al. [59] studied the influence of the powders’ properties on the tablets hardness and discovered a lot of the involved factors, proving the importance of using suitable materials in order to obtain tablets with good and reproducible mechanical resistance.

The specific parameter of the orally dispersible tablets is the disintegration time, which must be less than 3 min according to European Pharmacopoeia. Brniak W et al. [60] stated that the lyophilized ingredients are disintegrated in a matter of seconds in the oral cavity. In addition to the superdisintegrant role of the selected excipients, the use of the inclusion complexes obtained by lyophilization may also be an important factor that acted in shortening the disintegration time. Dobetti L. [61] affirmed that the disintegration behaviour of the orodispersible tablets prepared by direct compression depends on the distinct and mixed effects of the excipients used. Meanwhile, Moqbel H.A. et al. [62] declared that disintegration performance is given by both the disintegrants and the hydrophilic ingredients included in the formulation. The obtained results regarding the disintegration time proved a great selection of the inclusion complex based on the obtaining technique, adequate excipient choice and a well-conducted direct compression process. When adopting the lyophilization method for preparation, the NIF inclusion was at its highest, according to the results of the complexes’ physicochemical evaluation, indicating that these systems will have a better in vivo behaviour.

The influence of the cyclodextrin type is best highlighted in the in vitro studies, both disintegration and dissolution. It is proven that HP-β-CD ensures better in vitro performance of the tablets, but also the use of Me-β-CD in nifedipine tablets leads to satisfactory tablets for oral dispersion.

## 4. Materials and Methods

### 4.1. Materials

Nifedipine (NIF) was procured from Fagron (Thessaly, Greece), hydroxypropyl-β-cyclodextrin (HP-β-CD) and methyl-β-cyclodextrin (Me-β-CD) were obtained from Global Holding Group Co., Ltd., (Ningbo, China). F-MELT^®^ was acquired from Fuji Chemical Industries Co., Ltd. (Toyama, Japan), Disintequik™ ODT from Kerry Inc. (Beloit, WI, USA), PROSOLV^®^ SMCC HD 90 and EXPLOTAB^®^ were provided by JRS PHARMA GmbH & Co. KG (Rosenberg, Germany). LIGAMED^®^ MF-2-V was produced by Peter Graven (Venlo, NV, The Netherland). Chemicals and solvents used in this study were of analytical reagent grade.

The used compounds were weighed using a Mettler Toledo AT261 balance (sensitivity of 0.01 mg).

### 4.2. Methods

#### 4.2.1. Synthesis of the NIF-Cyclodextrins Binary Systems

Being A. et al. [19] performed the phase solubility test on the different molar ratios of NIF-Me-β-CD systems and it was proven that a 1:1 stoichiometric inclusion complex was apparent. Further, Gaspar de Araújo M.V. et al. [21] demonstrated that NIF solubility is highly increased by its complexation with HP-β-CD in the same 1:1 molar ratio. Based on the mentioned results, for the present study, a minimal 1:1 molar ratio of NIF-HP-β-CD and NIF-Me-β-CD was selected. As the aim of the study is to manufacture orodispersible tablets, in order to obtain adequate-weight pharmaceutical products, it is desirable to incorporate the lowest amount of inclusion complex.

To establish the proper technique which provides the higher complexation degree, the binary systems were synthesized by different techniques, both in the liquid and solid states. For characterization studies on complexes, a simple physical mixture (obtained using the same 1:1 molar ratio, after mixing the ingredients for 5 min, at room temperature) was used as a reference.

##### Synthesis Using Solid-State Kneading Technique

NIF and the two β-cyclodextrin derivatives (HP-β-CD and Me-β-CD) were brought into a mortar after accurate weighing, then kneaded with a low amount of ethanol:water (50:50 *v/v*) solution, for 1 h, at room temperature. The obtained wet adhesive mass was passed through a 12-mesh sieve and the granules were dried at 25 °C for 24 h before being ground into a fine powder.

##### Liquid-State Coprecipitation and Lyophilization Techniques

The ingredients were dissolved in the preferential solvent (NIF in ethanol and the two cyclodextrins in water). The NIF solution was progressively added to the CDs solutions and the resulting suspensions were stirred using a Heidolph MR 3001K magnetic stirrer for 6 h at 800 rpm and room temperature [63,64].

For the coprecipitation process, the resulting mixture was filtered, then the residue was dried for 24 h at 25 °C using an exicator.

In the case of the lyophilization method, the mixtures were frozen. For the lyophilization technique a Christ ALPHA 1–2, B Braun Biotech International (Melsungen, Germany) lyophilizer was used. The work conditions are, for the temperature, −60 °C and the time, 12 h.

#### 4.2.2. Inclusion Complexes Characterization

The Fourier Infrared spectra were recorded on an NICOLET 6700 FT-IR spectrophotometer (Thermo Electron Corporation, Waltham, MA, USA), in a range of 4000 to 400 cm^−1^. The FTIR spectra were obtained in transmittance mode from KBr pellets.

SEM micrographs were carried out by means of an FEI Quanta 3D FEG (Brno, Czech Republic) scanning electron microscope. The sample morphology was studied using a secondary electron detector at an acceleration voltage in a range of 5 to 20 kV, at different magnifications. The powders were placed on stub and scanned without special treatment.

XRD diffraction patterns were obtained using a Rigaku Ultima IV diffractometer in parallel beam geometry, with a CuKα radiation source (λ = 1.5406 Å) at 40 kV and 40 mA. The diffractograms were recorded from 5° to 60° at a step size of 0.02° and a speed of 2°/min.

DSC thermal curves were registered using a differential scanning DSC 8500 (Perkin-Elmer, Waltham, MA, USA) power-compensated calorimeter with a cooling system (Intracooler III). The used heating rate was 10 °C min^−1^. The experiments were made in inert atmosphere using dry nitrogen with a flow rate of 20 mL min^−1^. At the beginning of the heating program, samples were held for 2 min at 30 °C. Sealed aluminium pans of 20 μL volume were used in with an empty pan as reference. The DSC calorimeter was properly calibrated using high-purity indium as standard (*T*_fus_ = 156.7 °C and Δ*H*_fus_ = 28.5 J g^−1^), following the manufacturer’s specifications. The thermal effects were calculated using the Pyris Software for Windows version 13.4.0.0040 (Perkin-Elmer, Waltham, MA, USA). The DSC thermal curves were normalized to sample weight using Origin 7.5 Software.

#### 4.2.3. Preformulation Studies for the Orodispersible Tablets which Contain NIF-HP-β-CD and NIF-Me-β-CD Inclusion Complexes

Since the aim of the present study is to realize orally disintegrating tablets, direct compression is the ideal manufacturing method considering all of its benefits, especially the lack of humidity involved in the process, so the ingredients are well protected. It is crucial to develop a direct compression blend with good flowability and compressibility features in order to guarantee that the final tablets will display suitable pharmacotechnical and stability properties [65]. Considering the amorphous character of the lyophilization complex, obtaining blends with proper physical attributes for direct compression technology is very challenging and it requires an accurate selection of the excipients.

##### Powders for Direct Compression Formulation

All ingredients are calculated to obtain tablets with 300 mg weight and a dose of 10 mg nifedipine. Three formulations for each inclusion complex (NIF-HP-β-CD and NIF-Me-β-CD) were chosen and are shown in Table 6.

Three different formulations for each NIF inclusion complex were developed and the influence of the excipients on the powders’ properties was analysed. The variable excipients are Prosolv^®^ SMCC HD 90 (silicified microcrystalline cellulose) used as a binder agent, F-Melt^®^ (a spray-dried powder consisting of carbohydrates, inorganic ingredients and disintegrants) and Disintequik™ ODT (a co-processed excipient consisting of mannitol, dextrose monohydrate, monohydrate lactose and crospovidone), which are modern co-processed excipients, suitable for orodispersible tablets for their filling, glidant and disintegrating characteristics, together with a sweet and pleasant taste. For all formulations, Explotab^®^ (sodium starch glycolate) was chosen as superdisintegrating agent and Ligamed^®^ MF-2-V (magnesium stearate) was used for its lubricant ability.

##### Powders for Direct Compression Synthesis

The components were initially passed through a 20-mesh sieve, then weighed according to the established amounts. All ingredients, except the magnesium stearate, were mixed with 30 rpm speed, using a CMP 12 Plexiglas cube blender, from Pharmag GmbH (Klipphausen, Germany), for 30 min, at room temperature. In the end, the magnesium stearate was added and mixed for another 2 min under the same conditions.

##### Physical Analysis of the Powders

Fineness was established by analytical sieving, A CISA Sieve Shaker Mod. RP 10, obtained from Cisa Cedaceria Industrial (Barcelona, Spain) was used. The sieves are arranged in decreased order of fineness and 50 g of each powder is poured on the top sieve, then the system is subjected to vibrations with 800 rpm amplitude for 10 min. The powder retained on each sieve was gathered and weighed.

Flowability was evaluated by the flowing time, the angle of repose and the rate displayed by 60 g of each blend when it passed through an orifice with a standardized diameter. The tests were performed using an Automated Powder and Granulate Testing System PTG-S3, fabricated by Pharma Test Apparatebau GmbH (Hainburg, Germany).

Compressibility was assessed by determining the bulk and tapped density, Carr Index (CI) and Hausner ratio (HR), using the Vankel Tap Density Tester, produced by Vankel Industries Inc. (Austin, TX, USA). Further, 50 g of each formulation blend was poured into the apparatus cylinder, the bulk volume was measured, and the bulk density was calculated. The graduated cylinder containing the powder was subjected first to 500 mechanical shocks, then to another 750, each time reading the tapped volume. In the end, *HR* and *CI* were calculated according to the following equations:

(1) $$HR=\frac{\rho_{tapped}}{\rho_{bulk}}$$

(2) $$CI\left(\%\right)=100\times \frac{\left(\rho_{tapped}-\rho_{bulk}\right)}{\rho_{tapped}}$$

where *ρ_tapped_* is the tapped bulk density of the blend (kg/m^3^) and *ρ_bulk_* is the loose bulk density of the material (kg/m^3^). According to literature data, a *CI* value of less than 10 reveals a great flowability and compressibility for the material and an *HR* value below 1.25 points out a free flowing of the powder [66].

Moisture content was expressed as loss of drying registered using the thermogravimetric method, performed with an HR 73 Mettler Toledo halogen humidity analyser produced by Mettler-Toledo GmbH (Greifensee, Switzerland).

All analyses were performed five times for each formulation.

#### 4.2.4. Development and Manufacturing of the Orally Disintegrating Tablets with NIF-HP-β-CD and NIF-Me-β-CD Inclusion Complexes

##### Formulation of the Orodispersible Tablets

According to the results registered in the preformulation studies, F3 and F6, containing silicified microcrystalline cellulose and Disintequik™ ODT excipients, presented phamacotechnical properties adequate for direct compression process. Based on these remarks, the two materials were further processed as orodispersible tablets. The orally disintegrating tablet formulations are shown in Table 7.

##### Manufacturing Process of Orodispersible Tablets

The blends previously prepared as described above were compressed with a 35 kN compression force in a single-post eccentric Erweka EP-1 Tablet Press produced by Erweka, Germany. Further, 10 mm flat punches were used and the machine was set up to obtain tablets with 300 mg weight.

#### 4.2.5. Quality Control of the Orodispersible Tablets

##### Organoleptic Properties

The general appearance of the tablets was evaluated according to compendial specifications [48,67].

##### Dimensions (Diameter and Thickness)

The thickness and the diameter of ten tablets from each batch were measured using a VK 200 Tablet Hardness Tester, produced by Vanderkamp (Cleveland, NY, USA).

##### Mass Uniformity

Here, 20 tablets from each series were weighed separately, and the average weight was determined [48].

##### Hardness

The VK 200 Tablet Hardness Tester was used to measure the hardness. It is expressed as the load needed to crush the tablets placed between the two anvils in the apparatus. Hardness tests were repeated on ten tablets from each formulation.

##### Friability

The friability was determined on ten tablets from each batch using the Vankel friabilator. The tablets were weighed and placed in the apparatus drums, then subjected to 25 rpm for 5 min. The tablets are de-dusted and again weighed calculating the mass loss. It must not exceed 1.0% [48,67].

##### In Vitro Disintegration Time

The disintegration performance was assessed on six tablets from each series, using two different media: one obtained from distilled water at 37 ± 0.5 °C, according to compendial standards [32] and another one which is simulated saliva phosphate buffer with a pH = 6.8 at 37 ± 2 °C [68]. An Erweka DT 3 apparatus, produced by Erweka^®^ GmbH, Germany, was used and the time required for complete disintegration was registered in seconds.

##### In Vitro Dissolution Rate in Simulated Saliva Medium

The evaluation of dissolution behaviour was performed in a simulated saliva medium using the methodology described by Novac et al. [32]. Absorbance measurements were made on a UV-Vis Nicolet Evolution 100 spectrometer. The experiments were repeated six times for each tablet.

## 5. Conclusions

This work shows that nifedipine can form inclusion complexes with HP-β-CD and Me-β-CD, respectively, in a 1:1 molar ratio. The results obtained from FTIR, SEM, XRD and DSC studies showed that the solid NIF-HP-β-CD and NIF-Me-β-CD inclusion complexes can be obtained in a 1:1 molar ratio using kneading and lyophilization techniques. The obtained data establish that the lyophilization method can be an efficient and a promising method for the formation of inclusion complexes in the solid state, being, therefore, very attractive for the pharmaceutical domain. Finally, fast oral disintegrating nifedipine tablets were successfully prepared using the direct compression method. The thickness of the prepared tablets for the two formulations (F3 for NIF-HP-β-CD and F6 for NIF-Me-β-CD) was found to be ~4.00 mm. The friability of the compressed F3 and F6 tablets is within the range, i.e., less than 1%. The in vitro disintegration studies are found to be in 13 to 35 s for both F3 and F6 formulations and the in vitro dissolution is complete after 30 min in simulated saliva medium. Consequently, in the end, it was concluded that the prepared orodispersible tablets of inclusion complexes in nifedipine (10 mg) with HP-β-CD and Me-β-CD, respectively, prepared by the lyophilization method, may be a potential candidate for successful fast disintegrating tablet dosage form. In addition, the use of HP-β-CD for obtaining nifedipine inclusion compounds and further manufacturing of the orally disintegrating tablets was proved to show a better in vitro performance.

## Figures and Tables

**Table 1:** Wavenumbers (cm^−1^) of pure compounds NIF, HP-β-CD, their physical mixture and the inclusion complexes obtained using the three methods of complexation.

Assignment	NIF	HP-β-CD	NIF-HP-β-CD Physical Mixture	NIF-HP-β-CD Kneading Method	NIF-HP-β-CD Coprecipitation Method	NIF-HP-β-CD Lyophilization Method
N-H aromatic	3334				3330	
O-H stretching		3409	3428	3414		3435
C-H aliphatic	2960				2953	
C-H stretching		2925	2921	2924		2928
C=O ester	1682				1679	
O-H bending		1640	1652	1652		1653
NO_2_	1529		1557		1530	
C=C aromatic	1495					1436
C-C-O ester	1230				1227	
-C-O ester	1122				1120	
C-O vibration		1157		1153		1128
glycosidic bond		846				

**Table 2:** Wavenumbers (cm^−1^) of pure compounds NIF, Me-β-CD, their physical mixture and the obtained inclusion complexes obtained using the three techniques of complexation.

Assignment	NIF	Me-β-CD	NIF-Me-β-CD Physical Mixture	NIF-Me-β-CD Kneading Technique	NIF-Me-β-CD Coprecipitation Technique	NIF-Me-β-CD Lyophilization Technique
N-H aromatic	3334				3331	
O-H stretching		3419	3427	3428		3403
C-H aliphatic	2960				2953	
C-H stretching		2933	2931	2958		2932
C-H aliphatic		2840	2835	2897		2833
C=O ester	1682				1684	
O-H bending		1654	1684	1683		1684
NO_2_	1529					
C=C aromatic	1495					1456
C-C-O ester	1230					
-C-O ester	1122				1121	
C-O vibration		1157	1150			1159
C-OH groups		1029	1046	1045	1022	1043
glycosidic bond		858		845		

**Table 3:** Physical and volumetric characteristics in the direct compression powders.

Parameter	Formulation Code					
	F1	F2	F3	F4	F5	F6
Moisture content (%)	4.69 ± 1.24	5.13 ± 1.38	2.45 ± 0.79	4.85 ± 1.63	5.76 ± 2.25	2.79 ± 0.87
Flow time (s *	16.4 ± 0.96	18.2 ± 1.54	9.7 ± 0.65	15.3 ± 1.34	18.8 ± 1.33	10.3 ± 1.51
Angle of repose (θ degrees) *	34.22 ± 1.33	36.29 ± 2.55	28.14 ± 1.75	35.23 ± 1.86	37.45 ± 2.08	30.07 ± 1.12
Flow rate (g/s) *	3.658	3.296	6.185	3.921	3.191	5.825
Bulk density (g/mL)	0.562	0.585	0.518	0.558	0.574	0.536
Tapped density (g/mL)	0.663	0.699	0.567	0.692	0.703	0.591
Carr Index (CI) (%)	15.2	16.3	8.6	19.3	18.3	9.3
Hausner’s ratio (HR)	1.17	1.19	1.09	1.24	1.22	1.10

* no stirring, nozzle: 15 mm.

**Table 4:** Quality characteristics of the orally dispersible tablets.

Parameter	Formulation Code	
	F3	F6
Thickness (mm)	4.01 ± 0.21	4.02 ± 0.36
Diameter (mm)	10 ± 0.64	10 ± 0.57
Mass uniformity (mg)	302 ± 1.79	299 ± 2.45
Hardness (N)	55 ± 3.22	67 ± 4.16
Friability (%)	0.05 ± 0.04	0.11 ± 0.09
In vitro disintegration time—in water (seconds)	13 ± 1.25	21 ± 1.16
In vitro disintegration time—in simulated saliva (seconds)	26 ± 2.04	34 ± 1.83

**Table 5:** Dissolution behaviour of the orally dispersible tablets.

Time (min)	Dissolution Rate (%)	
	F3	F6
5	26.15 ± 1.29	18.57 ± 1.66
10	67.12 ± 0.93	53.44 ± 0.97
15	88.53 ± 1.32	77.26 ± 1.83
20	93.37 ± 2.08	89.33 ± 2.43
25	97.23 ± 0.65	95.32 ± 1.16
30	99.11 ± 1.84	98.79 ± 1.41

**Table 6:** The formulations of the direct compression blends containing NIF-HP-β-CD (F1, F2, F3) and NIF-Me-β-CD (F4, F5, F6) inclusion complexes.

Ingredients	Formulation/Amount (%)					
	F1	F2	F3	F4	F5	F6
NIF-HP-β-CD	18.17	18.17	18.17	-	-	-
NIF-Me-β-CD	-	-	-	15.94	15.94	15.94
PROSOLV^®^ SMCC HD 90—Silicified microcrystalline cellulose	48.30	-	68.83	51.06	-	71.06
F-MELT^®^	30.53	68.83	-	30.00	71.06	-
DISINTEQUIK™ ODT	-	10.00	10.00	-	10.00	10.00
EXPLOTAB^®^—Sodium starch glycolate	2.00	2.00	2.00	2.00	2.00	2.00
LIGAMED^®^ MF-2-V—Magnesium stearate	1.00	1.00	1.00	1.00	1.00	1.00

**Table 7:** The formulations of the orally dispersible tablets containing NIF-HP-β-CD (F3) and NIF-Me-β-CD (F6) inclusion complexes.

Ingredients	Quantity mg/Tablet		Role in Formulation
	F3	F6	
Inclusion complex NIF-HP-β-CD (1:1)	54.50	-	Active ingredient
Inclusion complex NIF-Me-β-CD (1:1)	-	47.82	Active ingredient
PROSOLV^®^ SMCC HD 90—Silicified microcrystalline cellulose	206.50	213.18	Filler—Binder
DISINTEQUIK™ ODT	30.00	30.00	Superdisintegrant
EXPLOTAB^®^—Sodium starch glycolate	6.00	6.00	Superdisintegrant
LIGAMED^®^ MF-2-V—Magnesium stearate	3.00	3.00	Lubricant
TOTAL	300.00	300.00	

## Data Availability

Data is contained within the article.
